# Intestinal Perforation in a patient with peritoneal carcinomatosis from colon cancer treated with Regorafenib. Description of a case and review of the literature

**DOI:** 10.1016/j.radcr.2024.02.018

**Published:** 2024-02-29

**Authors:** Maria Alessandra Bellia, Carmelo Sofia, Maria Adele Marino, Carmelo Mazzeo, Santino Antonio Biondo, Eugenio Cucinotta, Francesco Fleres

**Affiliations:** a Section of Radiological Sciences, Department of Biomedical Sciences and Morphological and Functional Imaging, University of Messina, Policlinico “G. Martino” Via Consolare Valeria 1, 98125, Messina, Italy; b Department of Human Pathology of the Adult and Evolutive Age “Gaetano Barresi”, Section of General Surgery, University of Messina, Via Consolare Valeria, 98125, Messina, Italy

**Keywords:** Colon cancer, Peritoneal metastases, Intestinal perforation, Regorafenib, Chemotherapy induced adverse event

## Abstract

Regorafenib is a multikinase inhibitor approved for treatment of patients with metastatic Colo-Rectal Cancer (mCRC) and Gastro-Intestinal Stromal Tumor (GIST) progression after the administration of other tyrosine-kinase inhibitors such as imatinib and sunitinib.

Only a handful of severe side effects such as intestinal perforations and fistulas have been described in the literature in patients undergoing multikinase inhibitor treatment. We report a case of a patient with peritoneal mCRC who experienced an intestinal perforation during the administration of Regorafenib and review the literature. A 48-year-old man with previously resected sigmoid colon cancer and peritoneal metastatic disease under Regorafenib treatment presented to our Emergency Department with severe abdominal pain and asthenia. Abdominal X-ray and contrast-enhanced computed tomography examination revealed an intestinal perforation. The patient underwent emergency surgery which demonstrated acute diffuse peritonitis, necrosis, and perforation of a distal ileal loop affected by peritoneal metastatic disease. The necrosis of peritoneal implants on bowel walls could be regarded as a potential factor leading to intestinal perforation in metastatic colorectal cancer patients undergoing Regorafenib treatment complaining of severe abdominal pain and asthenia.

Surgeons, radiologists and oncologists should always keep in mind this rare adverse event during Regorafenib administration. Appropriate diagnostic tests and treatments should be carried out.

## Introduction

Regorafenib is a multikinase inhibitor acting on a wide spectrum of tyrosine kinase targets involved in regulating angiogenesis and tumor cell proliferation, and inhibiting intracellular signaling to affect the tumor micro environment and mutant oncogenetic kinases KIT, RET, and B-RAF [1,2].

Regorafenib is approved for treatment of patients with metastatic colorectal cancer (mCRC) and gastrointestinal stromal tumor (GIST) progression following prior administration of other tyrosine-kinase inhibitors such as imatinib and sunitinib [2], [3], [4], [5], [6].

Common side effects of Regorafenib include dermatologic toxicity such as hand-foot skin reaction and severe rash, hypertension, asthenia, liver function abnormalities, infections, hemorrhage, and diarrhea.

Only a few serious side effects such as intestinal perforations and fistulas have been described in the literature. We report a case of a patient with peritoneal mCRC who developed an intestinal perforation during the administration of Regorafenib and review the literature.

## Case report

A 48-year-old man was admitted to the Emergency Department of our hospital for asthenia and acute abdominal pain. Two years prior, he underwent surgical treatment for sigmoid colon cancer, followed by 12 cycles of chemotherapy (FOLFOX) a year later due to the development of peritoneal carcinomatosis.

Three weeks prior he was discharged from another medical center with a diagnosis of persistent peritoneal metastatic disease originating from colon cancer and a treatment regimen comprising of 2 courses of Regorafenib.

**Fig. 1:**
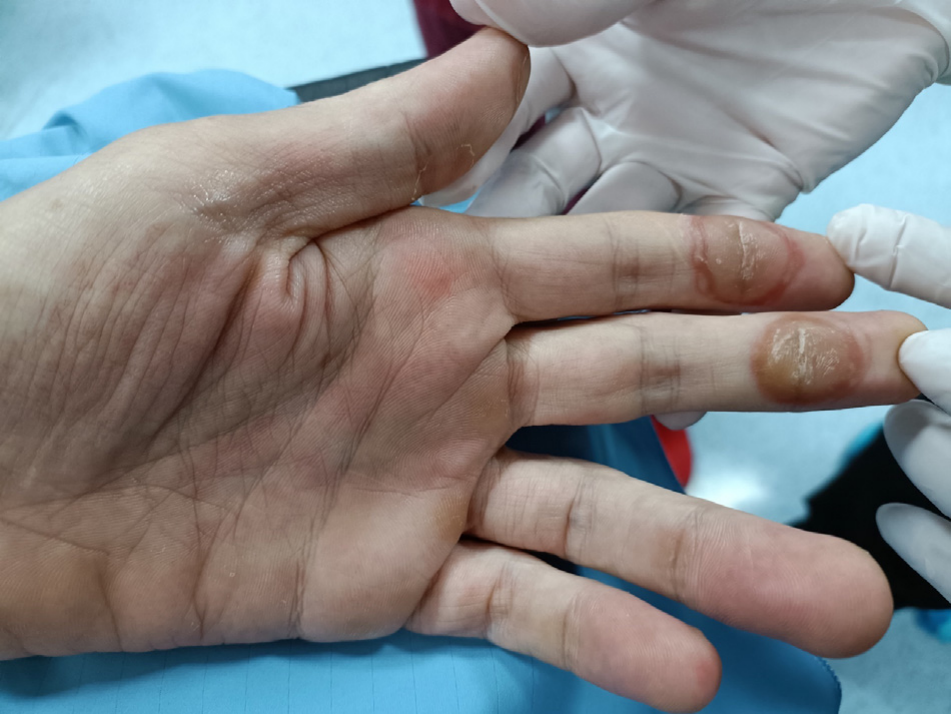
Hand-skin reaction characterized by focal callus-like hyperkerotoses with peeling and oedema.

The treatment protocol of the patient involved a 28-day course of Regorafenib (4 160-mg daily tablets for 21 days), but chemotherapy had to be discontinued after 14 days of treatment due to the development of severe skin alterations on the hands (hand-foot skin reaction) (Fig. 1) along with severe abdominal pain.

Laboratory tests were performed, and results were as follow:

 white blood cells [WBC]: 4.100 mm^2^ (85% neutrophils); red blood cells [RDB]: 3.740.000 mm^2^; direct bilirubin: 2.42 mg/dL [*0-0.30]; total bilirubin: 5.16 mg/dL [*0-1.2]; amylase: 1452 U/L [*0-100]; lipase: 8416 U/L [*8-57]; markers CA 19.9. 1654.53 [* <37]; CA 125: 76 UI/mL [* <30]. Negative Covid swab.

**Fig. 2:**
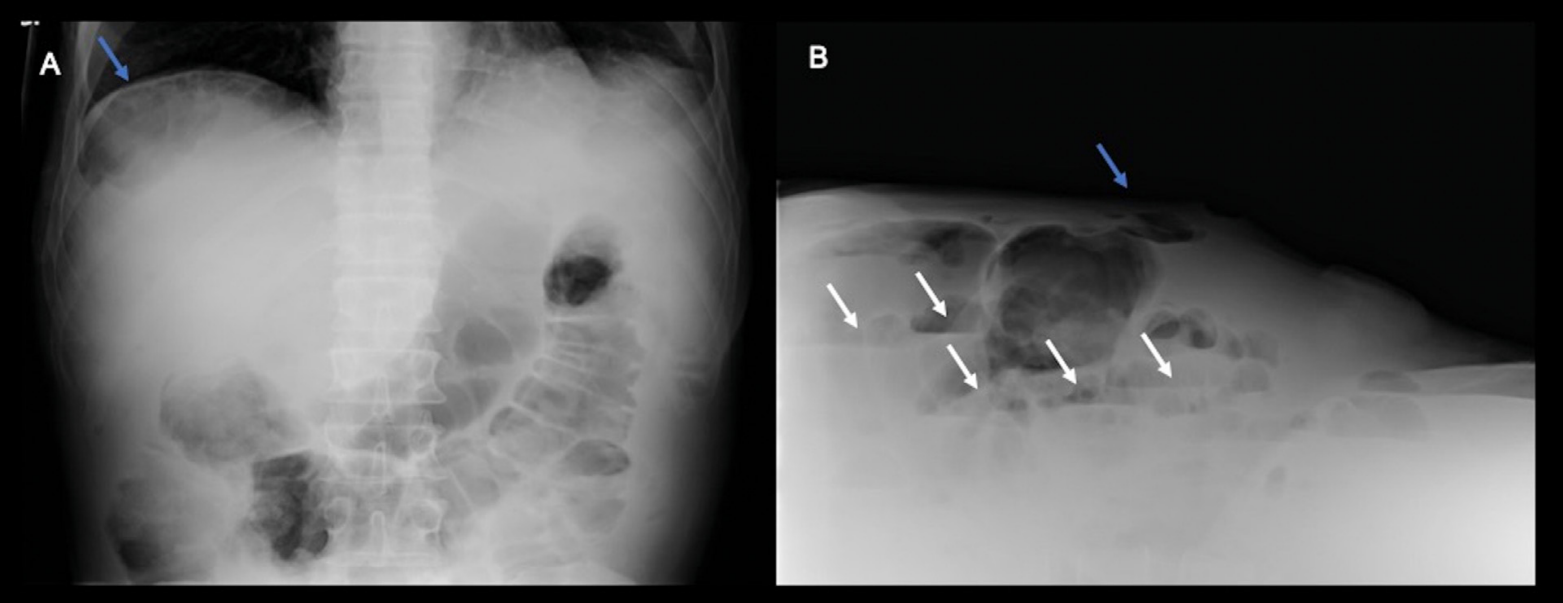
Supine frontal and cross-table lateral view radiographs of the abdomen. Free gas (blue arrows) is seen beneath the right hemidiaphragm on the frontal view and in the non-dependent anterior abdominal peritoneal space on the cross-table lateral view. Also note gas distension of the stomach, duodenum and bowel loops, most of them presenting air-fluid levels (white arrows).

The patient underwent an X-ray of the abdomen (Fig. 2A and B) that revealed gas distension of the stomach, duodenum and bowel loops, most of them presenting air-fluid levels, and free gas bubbles in ante dependent position as for pneumoperitoneum. The patient started vomiting and therefore a nasogastric tube was placed for decompression.

**Fig. 3:**
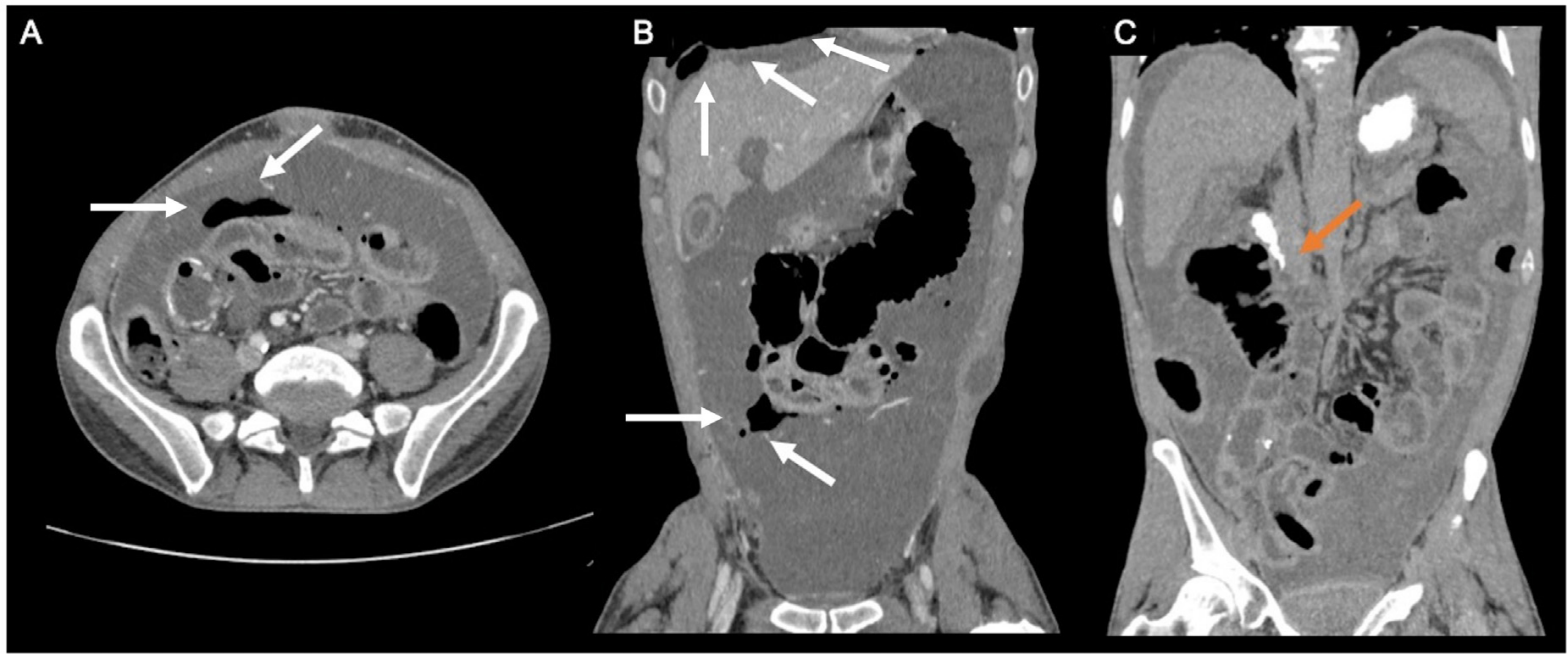
Contrast enhanced CT scan of the abdomen and pelvis, on axial (A) and coronal (B-C) planes, the latter (C) after oral administration of iodinated oral contrast. The pictures show massive neoplastic ascites and pneumoperitoneum (white arrows). On C no progression of the iodinated oral contrast is seen beyond the second portion of the duodenum (arrow).

A following contrast-enhanced CT scan of the abdomen and pelvis (Fig. 3A-C) revealed extensive ascites, widespread peritoneal metastatic disease affecting the surface of the small bowel loops and confirmed the presence of free intraperitoneal gas as for intestinal perforation close to a distal ileal loop. After the administration of an oral water-soluble iodinated contrast agent, no progression of contrast material beyond the second duodenal portion was obtained, due to the intestinal encasement related to the peritoneal disease (Fig. 3C).

**Fig. 4:**
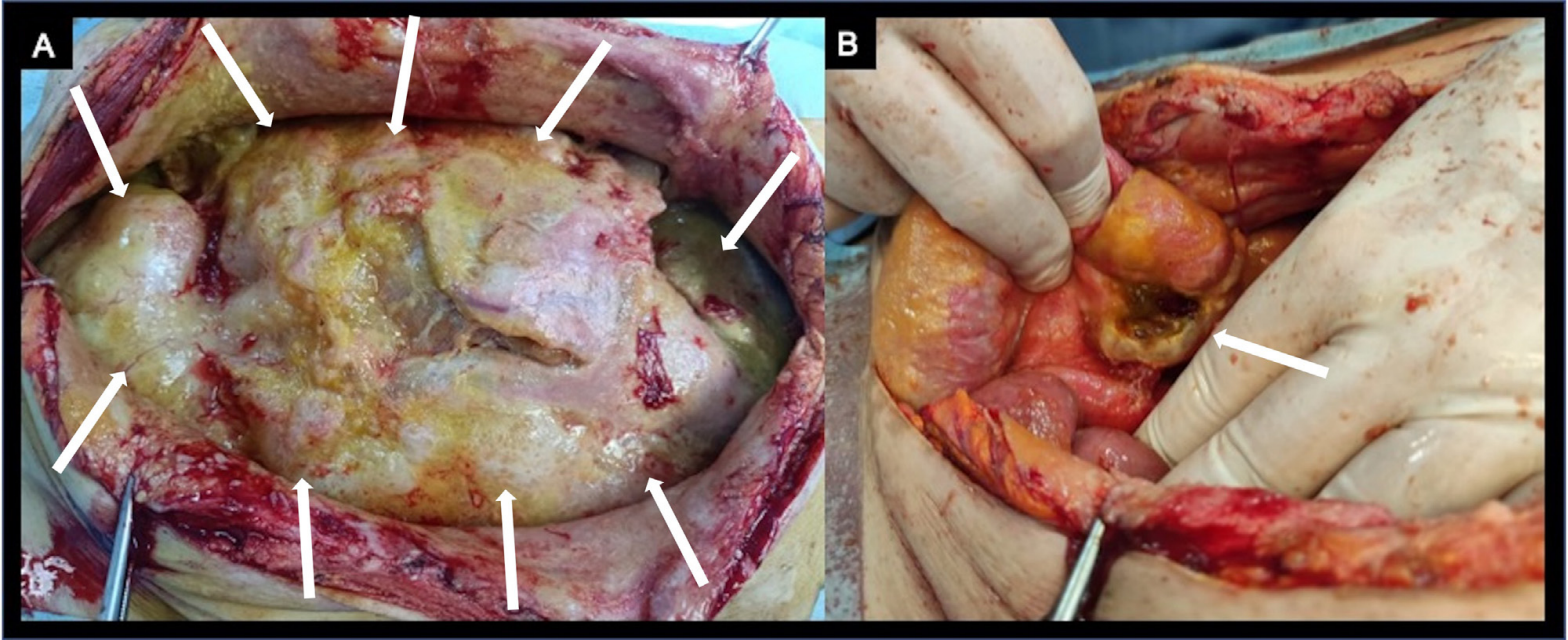
Pictures from the surgical room showing diffuse peritoneal metastatic disease (arrows, A) and the site of the intestinal necrosis and perforation (arrow, B).

Given the patient's compromised health conditions, a conservative approach was chosen. Initially, percutaneous drainage was implemented; however, within 24 hours, it revealed a significant output rate (approximately 1 liter) of enteric fluid, prompting consideration for surgical exploration. Thus, the patient underwent an exploratory laparotomy demonstrating diffuse peritoneal carcinomatosis (Fig. 4A) and acute peritonitis with necrosis and perforation of the terminal ileum wall (Fig. 4B) at a distance of 7 cm from the ileocecal valve, in correspondence of a peritoneal implant over the intestinal surface. A resection of the perforated tract of the ileum and then a terminal ileostomy was performed. Due to the retraction of the right mesocolon, the cecostomy couldn't be exteriorized and it could not be left in the abdomen after mechanical closure, due to the high risk of leakage for “high pressure” related to the multiple nodules of carcinosis occluding the remaining part of the colon. Therefore, a cecostomy was successfully performed using a PEG-Tube instead of a Pezzer probe, as the latter was unavailable during the surgical operation, to exteriorize the enteric material.

Two weeks later, the patient was discharged, and upon returning for outpatient follow-up 2 months later, he exhibited stable medical conditions, leading to the removal of the PEG-Tube.

## Discussion

Managing patients with metastatic colorectal cancer remains a formidable challenge for medical oncologists. The majority of these patients present with unresectable tumors, and the most impactful treatment options often involve second- or third-line chemotherapy.

In September 2012, the Food and Drug Administration (FDA) approved Regorafenib as a salvage treatment for patients having metastatic colorectal cancer (mCRC), who were previously treated with or without anti-VEGF or anti-EGFR therapy and for patients with standard chemotherapy-resistant gastrointestinal stromal tumors (GISTs)*.* In 2013 Regorafenib gained approval for treating refractory advanced colorectal cancer and advanced gastrointestinal stromal tumors (GIST) following failure of at least imatinib and sunitinib treatment [2,[4], [5], [6].

The most common adverse events of grade three or higher related to Regorafenib were hand-foot skin reaction, fatigue, diarrhoea, hypertension, and rash or desquamation [3], [4], [5], [6], [7]. There are criteria for dose reduction and disruption which are set for every side effect in such cases.

Gastrointestinal perforation and fistula are rare serious adverse events and only a very few cases have been reported in the literature so far (Table 1). If, during the administration of the drug, these events occur, clinical studies and FDA warnings and precautions suggest its immediate discontinuation [2,[4], [5], [6], [7], [8], [9].

**Table 1:** Summary of regorafenib-related intestinal fistulas and perforations reported in literature with references.

Authors (y)	Age (y) sex	Tumor	Treatment for primary tumor	Symptoms	Onset (d)	Imaging (CT/ X-ray)	Treatment for intestinal perforation	Outcome
Adenis et al. [14] 2013	66 M	GIST with peritoneal metastases	Gastrectomy Imatinib and Sunitinib	Abdominal pain and tenderness	58	Sign of peritonitis and an ileal necrotic mass	Conservative	Septic shock, died 24 hours after the admission
Adeniset al. [14] 2013	72 F	Colon Cancer and peritoneal carcinomatosis	Surgery FOLFIRI + cetuximab	Abdominal pain and tenderness	7	Entero-cutaneous fistula	Discontinue regorafenib	Died of disease progression four months later
Ogataet al. [3] 2016	65 F	Cecal cancer with hepatic and lymph node metastases	FOLFOX+Bevacizumab FOLFIRI+Bevacizumab	Abdominal pain and tenderness	32	Free air outside of the cecal tumor	Emergency surgery, ileocecal resection and colostomy	Surgical site infection; discharged after thirty days
Doi et al. [15] 2016	78 F	Ascending colon cancer with peritoneal metastases	Surgery FOLFOX+Bevacizumab FOLFIRI+Bevacizumab	Abdominal pain	10	Perforation of the jejunum involved by peritoneal metastases	Surgery	Improved
Doi et al. [15] 2016	62 M	Rectal cancer	Surgery FOLFOX+Bevacizumab FOLFIRI+Bevacizumab	Fever and abdominal pain	51	Abscess and Fournier's gangrene due to the rectal-perineal fistula	Surgery	Improved
Doi et al. [15] 2016	59 M	Rectal cancer	Surgery; radiation FOLFOX+Bevacizumab FOLFIRI + Bevacizumab	Fever and abdominal pain	20	Abscess and Fournier's gangrene due to fistula	Surgery	Improved
Doi et al. [15] 2016	44 M	Rectal cancer	FOLFOX/Bevacizumab FOLFIRI + Aflibercept	Fever and abdominal pain	84	Abscess and Fournier's gangrene due to fistula	Surgery	Improved
Doi et al. [15] 2016	52 M	Transverse colon cancer	FOLFOX+Bevacizumab FOLFIRI+Bevacizumab	Fever	5	Fistula	Conservative	Improved
Sarici et al. [7] 2018	60 F	Colon adenocarcinoma with liver and peritoneal metastases	Hemicolectomy FOLFOX FOLFIRI+Bevacizumab	Abdominal pain and tenderness	9	Free air in the abdomen, suspected perforation	Emergent surgery for perforation next to the ileocolic anastomosis	No serious complication, wound infection
Ouchi et al. [3] 2021	60 M	Cecal cancer with liver and peritoneal metastases	Ileo-trasversostomy FOLFOX+Bevacizumab FOLFIRI	Severe pain around the navel	7	Free air due to perforation of the anastomotic site and ascites	Conservative	Died thirty days after the discharge

The incidence of intestinal perforation and fistula was approximately 0.6% in a cohort of 4518 patients treated with Regorafenib across various clinical trials when administered as a single agent [5]. In contrast, for other drugs targeting VEGF pathways, such as bevacizumab, the occurrence of gastrointestinal perforation ranged from 0.3% to 2.4% in clinical studies [10].

Intestinal perforation has been described in 4 patients with colon cancer and 1 with GIST, whereas entero-cutaneous fistulas occurred in five patients having colon cancer. In 6 cases the primary tumor was surgically removed. These incidents occurred in individuals with metastatic cancer who had previously undergone standard chemotherapy or monoclonal antibody treatment, typically the primary choice in managing metastatic colorectal cancer (mCRC) and GIST [6,[11], [12], [13], [14], [15], [16]. All patients at the CT scan reported metastases in lymph nodes or liver or peritoneal carcinomatosis. After the diagnosis of intestinal perforation, four patients underwent conservative treatment, while emergency surgery was performed in 6 patients, mirroring our case. Common signs and symptoms included abdominal pain, fever, and abdominal tenderness. Imaging, particularly CT scans, played a crucial role in diagnosing perforation by revealing free air in the abdominal cavity near the site of the perforation and identifying fistulas. In our case, CT demonstrated pneumoperitoneum and free air proximal to the site of intestinal wall disruption.

Outcomes varied, with 3 patients succumbing to the condition, while 7 successfully survived.

Regorafenib exerts its effects by diminishing multikinase activity in tumor cells, resulting in the impairment of vascular endothelial cell function [3,^8^,[17], [18], [19]. The suggested mechanism for intestinal perforation due to VEGF inhibitors involves the reduction of capillary density in the intestinal mucosa, leading to compromised wound healing and fistula formation. This process is further compounded by the shrinkage and necrosis of tumors infiltrating the intestinal mucosa, as well as bowel ischemia caused by thrombosis, ultimately resulting in perforation [8,^18^,[20], [21], [22]. It has been documented that gastrointestinal obstruction, arising from the presence of a tumor mass invading the bowel wall and peritoneal metastases, is a predisposing condition for intestinal perforation in patients treated with angiogenesis-targeting agents, such as Regorafenib [17,[19], [20].

Typically, perforations induced by anti-VEGF drugs such as Regorafenib occur in proximity to the original tumor site [3,18] or the anastomotic site [4,^8^,[16], [17], [18],21]. However, in our case, the perforation site did not align with the primary tumor or the anastomosis, a scenario documented in only one other case in the literature. This deviation was likely associated with the necrosis of a peritoneal implant affecting the wall of the distal small bowel [18].

The reported elapsed time from the initiation of regorafenib use to intestinal perforation varies between 5 and 84 days [2,^3^,^18^,19]. In our patient, gastrointestinal perforation occurred within 14 days of starting regorafenib, with an assumed cumulative dose of 160 mg over this period.

The onset of nonspecific symptoms such as abdominal pain, fever, and asthenia in a patient with a history of colon cancer or gastrointestinal stromal tumor undergoing Regorafenib treatment should promptly raise suspicion of possible intestinal perforation. Immediate discontinuation of the drug is recommended, and conducting diagnostic tests promptly can alter the potentially fatal outcome and prevent complications.

## Conclusions

The emergence of abdominal pain in metastatic colon cancer patients receiving Regorafenib raises suspicion of potential gastrointestinal perforation, necessitating thorough imaging and consideration of surgical interventions. Necrosis of peritoneal metastases involving intestinal walls might predispose such patients to intestinal perforation.

Our case presents several noteworthy aspects. Firstly, it underscores the importance of recognizing this uncommon adverse event, requiring heightened awareness among surgeons, radiologists, and oncologists. Secondly, it highlights the considerable challenges associated with the treatment, which is currently not well-defined. In certain cases, percutaneous drainage of abdominal collections with antibiotics has shown favorable outcomes based on patient conditions. Conversely, some instances necessitated surgical exploration. In our case, initially opting for a conservative approach due to the patient's compromised health, the multidisciplinary team initiated percutaneous drainage. However, within 24 hours, the drainage revealed a high output rate (approximately 1 liter) of enteric fluid, prompting a subsequent surgical exploration. In the context of damage control surgery, a PEG probe serves as a viable alternative to a Pezzer Tube.

Prompt recognition and appropriate timely treatment of perforation in metastatic colon cancer patients undergoing Regorafenib may result in improved patient outcomes.

## Authors’ contribution

Sofia C and Fleres F participated in the conception and design of the report. Sofia C, Fleres F, Bellia MA, and Marino MA drafted the paper and analyzed the report. Fleres F and Mazzeo C performed the surgical procedure. Fleres F, Mazzeo C, Biondo SA, and Cucinotta E have been involved in the diagnosis, surgical management and follow-up of the patient, and revised the text.

## Ethical approval

The authors declare that all the procedures followed comply with the ethical standards of the committee on human experimentation (both institutional and national) and with the Helsinki Declaration of 1975, as revised in 2013. Informed consent was obtained from the patients for being included in the study.

## Authors’ responsibility

The authors confirm: 1) that they have not previously published or submitted the same manuscript elsewhere, 2) that they have played a significant part in drafting this paper and approved the final version of the manuscript, 3) that they have complied with ethical standards, 4) that they have obtained all the necessary permissions to publish the figures and tables in the manuscript.

## Patient consent

Written informed consent was obtained from the patients ahead of the publication.
